# Role of Peroxisome Proliferator-Activated Receptor *γ* in Ocular Diseases

**DOI:** 10.1155/2015/275435

**Published:** 2015-06-04

**Authors:** Su Zhang, Hongwei Gu, Nan Hu

**Affiliations:** Department of Ophthalmology, Affiliated Hospital of Nantong University, Nantong, Jiangsu 226001, China

## Abstract

Peroxisome proliferator-activated receptor *γ* (PPAR *γ*), a member of the nuclear receptor superfamily, is a ligand-activated transcription factor that plays an important role in the control of a variety of physiological processes. The last decade has witnessed an increasing interest for the role played by the agonists of PPAR *γ* in antiangiogenesis, antifibrosis, anti-inflammation effects and in controlling oxidative stress response in various organs. As the pathologic mechanisms of major blinding diseases, such as age-related macular degeneration (AMD), diabetic retinopathy (DR), keratitis, and optic neuropathy, often involve neoangiogenesis and inflammation- and oxidative stress-mediated cell death, evidences are accumulating on the potential benefits of PPAR *γ* to improve or prevent these vision threatening eye diseases. In this paper we describe what is known about the role of PPAR *γ* in the ocular pathophysiological processes and PPAR *γ* agonists as novel adjuvants in the treatment of eye diseases.

## 1. Introduction

Peroxisome proliferator-activated receptor *γ* (PPAR *γ*), a member of the nuclear receptor superfamily, is a ligand-activated transcription factor that plays an important role in the control of gene expression linked to a variety of physiological processes [1]. PPAR *γ* was first identified by homology cloning in* Xenopus* [2] and then in mammals [3] and mice [4]. It is most widely expressed in adipose tissue but is also expressed in immune/inflammatory cells (e.g., monocytes, macrophages), mucosa of the colon and cecum, skeletal muscle, heart, kidney, liver, lung, and the eye ball [5–8]. Similar to typical nuclear receptors, PPAR *γ* is comprised of distinct functional domains, including an N-terminal transactivation domain (AF1), a highly conserved DNA-binding domain (DBD), and a C-terminal ligand-binding domain (LBD) containing a ligand-dependent transactivation function (AF2) [9].

PPAR *γ* can regulate transcription by several mechanisms, including ligand-dependent transactivation, ligand-dependent transrepression, and ligand-independent repression. PPAR *γ* is activated by heterodimerization with the retinoid X receptor (RXR) into biologically active transcription factor and then binds to peroxisome proliferator response elements (PPREs), thereby acting as a transcriptional regulator [10, 11]. PPAR *γ* is also capable of regulating gene expression independently of binding to PPREs. PPAR *γ* possesses a large T-shaped ligand-binding pocket that enables interaction with a structurally diverse library of ligands [12]. A wide range of natural and synthetic compounds functioning as PPAR *γ* ligands have been identified (Table 1).

Endogenous ligands for PPAR *γ* include unsaturated and oxidized fatty acids, nitrated fatty acids, eicosanoids, and prostaglandins [13]. Thiazolidinediones (TZDs) including troglitazone, pioglitazone, and rosiglitazone are synthetic PPAR *γ* ligands with the efficacy to enhance insulin sensitivity in animals and humans [14, 15], and some of the TZDs are already in clinical use as insulin sensitizers in type 2 diabetic patients [16]. With more intensive investigations, the number of PPAR *γ* ligands continues to increase for their key role in regulating metabolic processes.

PPAR *γ* is involved in a range of distinct physiological processes including fat cell differentiation, glucose homeostasis, lipid metabolism, aging, and inflammatory and immune responses [1, 17–20]. Previous investigations have found that PPAR *γ* and its ligands have good antiangiogenesis and antifibrosis effects in various organs [21–24]. Furthermore, recent studies indicate that PPAR *γ* plays an important role in oxidative stress response. It may directly modulate activation of several antioxidants involved in oxidative stress and influence apoptotic or necrotic cell death [25]. In regard to the immune system, PPAR *γ* is found in monocytes, macrophages, T cells, and dendritic cells and has been identified as crucial regulator of inflammatory gene expression [26–31]. As the pathologic mechanisms of major blinding diseases, such as age-related macular degeneration (AMD), diabetic retinopathy (DR), keratitis, and optic neuropathy, often involve neoangiogenesis and inflammation- and oxidative stress-mediated cell death, evidences are accumulating on the potential benefits of PPAR *γ* to improve or prevent these vision threatening eye diseases. However, there also several studies that reported the side effects of PPAR *γ* in some ocular diseases [32–37]. In this paper we describe what is known about the role of PPAR *γ* in the ocular pathophysiological processes and PPAR *γ* agonists as novel adjuvants in the treatment of eye diseases.

## 2. PPAR ***γ*** and Ocular Disease

### 2.1. PPAR *γ* and Ocular Surface Disease

#### 2.1.1. PPAR *γ* and Corneal Neovascularization and Fibrosis

The cornea is an avascular tissue and must remain transparent to refract light properly. Corneal neovascularization and fibrosis often lead to loss of corneal transparency which is an important cause of blindness. Diseases associated with corneal neovascularization include inflammatory disorders, corneal graft rejection, infectious keratitis, traumatic and chemical insults, contact lens-related hypoxia, aniridia, and limbal stem cell deficiency [38]. As potential angiogenesis modulators [39, 40], PPAR *γ* ligands have a good inhibition of corneal neovascularization [6, 41–43]. In 1999, Xin et al. [6] first reported that administration of 15d-PGJ2 inhibited vascular endothelial cell growth factor- (VEGF-) induced angiogenesis in the rat cornea. Then, Usui et al. [41] found that telmisartan, a partial agonist of PPAR *γ*, significantly reduced vascularized area in mice cornea. Furthermore, telmisartan-induced inhibition of corneal neovascularization was partially reversed by the administration of GW9662 (a PPAR *γ* antagonist), indicating that the inhibitory effects were partially mediated through PPAR *γ* signaling. Sarayba et al. [43] randomly divide twenty-six adult male Sprague-Dawley rats into three groups. Each group received intrastromal polymer micropellets containing different doses and types of pioglitazone. The area and density of neovascularization were measured 7 days after pellet implantation. The result indicated that pioglitazone can effectively inhibit VEGF-induced corneal neovascularization. Recently, Uchiyama et al. [42] also demonstrated that the ophthalmic solution of the PPAR *γ* agonist could inhibit inflammation, decrease the fibrotic reaction, and prevent neovascularization in the cornea from the early phase after alkali burn injury. Corneal neovascularization is a complex process that includes degradation of basement membrane and proliferation, migration, and tube formation by endothelial cells [44]. Activation of PPAR *γ* suppresses endothelial cell differentiation into tube-like structures and represses growth factor-induced endothelial cell proliferation in vitro [6]. PPAR *γ* activation also inhibits the expression of at least three important genes in the angiogenic process, the VEGF receptors Flk/KDR, Flt-1, and the protease uPA [6]. In addition, PPAR *γ* can reduce the activity of angiogenesis by inhibiting inflammatory cytokines at the transcriptional level via suppression of the AP-1, NF-*κ*B pathway [45]. These observations indicate some possible molecular mechanisms by which PPAR *γ* ligands mediate inhibition of corneal neovascularization. The TGF*β*-induced differentiation of corneal keratocytes into myofibroblasts plays a critical role in corneal scarring. PPAR *γ* ligands have antifibrotic effects and have been studied as agents capable of inhibiting TGF*β*-induced myofibroblast differentiation in cornea [46–49]. Using corneal fibroblasts cultured in vitro, Huxlin et al. [46] and Pan et al. [48] demonstrated that pioglitazone suppressed TGF*β*-induced alpha smooth muscle actin (*α*SMA) expression, inhibited cell migration, contractility, matrix metalloproteinase (MMP) secretion, and extracellular matrix production. Electrophilic PPAR *γ* ligands, CDDO-Me and *κ*15d-PGJ2, were also able to inhibit corneal fibroblast to myofibroblast differentiation in vitro [47]. Viral transfection and overexpression of PPAR *γ* inhibited activation of ocular fibroblasts and macrophages in vitro and also reduced myofibroblast differentiation, upregulation of several cytokines and matrix metalloproteases, and macrophage/monocyte invasion in an alkali-burned mouse cornea [50]. Since viral transfection is not yet widely practiced clinically, several authors investigated the effects of topical PPAR *γ* ligands on corneal fibrosis [42, 46]. Topical application of rosiglitazone to cat eyes following laser ablation of the corneal stroma decreased *α*SMA expression, blocking myofibroblast differentiation, while allowing the epithelium and stroma to return to a normal thickness, restoring corneal shape, structure, and optical quality to near-normal levels [46]. The ophthalmic solution containing 0.1% pioglitazone hydrochloride significantly decreased the fibrotic reaction in the rat cornea after alkali burn injury [42]. All these evidences suggest that PPAR *γ* ligands may exert a direct antifibrosis effect to prevent the corneal scar formation.

#### 2.1.2. PPAR *γ* and Conjunctiva Fibrosis

Conjunctival scarring potentially reduces filtration efficacy after glaucoma filtering surgery. Yamanaka and his fellows [51] found that PPAR *γ* gene transfer suppresses the fibrogenic reaction in human subconjunctival fibroblasts (hSCFs) as well as the injury-induced scarring of conjunctival tissue in mice. PPAR *γ* overexpression may reduce the expression of type I collagen, fibronectin, and connective tissue growth factor (CTGF) in cultured hSCFs. It may also suppress invasion of macrophages into the healing subconjunctival tissue and generation of myofibroblasts [51]. Consistent with this result, Fan [52] and his fellows reported that rosiglitazone can effectively attenuate activation of human Tenon's fibroblasts (HTFs) induced by TGF*β*1 without obvious toxicity. The possible mechanism might be that rosiglitazone interferes with TGF*β*/Smad signaling pathway. Thus, PPAR *γ* and its agonists may represent a new strategy for inhibiting excessive bleb scarring in the conjunctiva after glaucoma surgery. Pterygium is a wing-like fibrovascular proliferation, of exposed bulbar conjunctival tissue, which encroaches onto the cornea. PPAR *γ* is positively expressed in pterygium specimens obtained from patients undergoing routine pterygium excision [53]. The role of PPAR *γ* as a potential therapeutic agent for pterygium was studied on cultured human pterygium fibroblasts (HPFs) in vitro. The results showed that PPAR *γ* agonists can significantly inhibit HPFs proliferation and induce apoptosis of HPFs in dose- and time-dependent manners [53, 54].

#### 2.1.3. PPAR *γ* and Dry Eye

Recently, Chen et al. [55] reported that the PPAR *γ* expression in the conjunctiva of dry eye mice was downregulated, accompanied by increased contents of inflammatory cytokines, TNF-*α* and IL-1*β*. They also found that pioglitazone may activate PPAR *γ* to suppress the inflammatory progression, increase the tear fluid production, elevate the tear film stability, and reduce the damage to the ocular surface, exerting a therapeutic effect on dry eye. In cultured lacrimal gland acinar cells, pioglitazone can inhibit NO production, excessive synthesis of which may be detrimental to normal function of the lacrimal gland, suggesting that the use of PPAR *γ* agonist may provide an effective therapeutic intervention for the prevention of dry eye caused by decrease or lack of lacrimal gland secretion [56].

#### 2.1.4. PPAR *γ* and Meibomian Gland Dysfunction (MGD)

Recently, Jester and Nien published a series of papers on PPAR *γ* and MGD [7, 57, 58]. Their studies have shown that mouse and human meibomian glands undergo specific age-related changes, including decreased acinar cell proliferation, decreased meibomian gland size, and increased inflammatory cell infiltration. These changes occur concurrently with altered PPAR *γ* localization from cytoplasmic-vesicular/nuclei of acinar cells in young mice and humans to nuclei in older individuals. Meibomian glands express PPAR *γ* in lipid synthesizing cells and PPAR *γ* is a biomolecular marker for meibocyte differentiation. By analyzing eyelid tissue from 36 patients (age range, 18–95 years) who underwent canthoplasty procedures, they found that the degree of MGD dropout was significantly correlated with nuclear PPAR *γ* staining, indicating that age-related MGD may involve altered PPAR *γ* localization. Based on these findings, Jester et al. proposed that age-related MGD involves altered regulation of PPAR *γ* gene that may lead to decreased meibocyte differentiation, acinar atrophy, decreased lipid synthesis, and the development of hyposecretory MGD.

### 2.2. PPAR *γ* and Retinal Disease

#### 2.2.1. PPAR *γ* and AMD

Age-related macular degeneration (AMD) is a degenerative disease of the macula which results primarily in loss of central vision [59]. The disease can be classified into a dry or nonexudative form (geographic atrophy) and a wet or exudative form (neovascular AMD). There is a growing body of research demonstrating that PPAR *γ* may be involved in various chemical pathways associated with AMD. PPAR *γ* is constitutively expressed in normal neuroretina and retinal pigmented epithelial (RPE) cells of mice and humans. However, the expression of PPAR *γ* is significantly higher than normal in both Ccl2^−/−^/Cx3cr1^−/−^ mice (an AMD model) and AMD patients [60]. The exudative form of AMD, characterized by choroidal neovascularization (CNV), is thought to be responsible for most of the cases of severe visual loss in this disease. Murata et al. [61] have demonstrated that PPAR *γ* ligands, troglitazone or rosiglitazone, significantly inhibited VEGF-induced proliferation and migration of RPE and choroidal endothelial cells and choroidal angiogenesis in vitro. In the eyes of rat and monkeys in which CNV was induced by laser photocoagulation, this group also showed that intravitreal injection of troglitazone dramatically inhibited the percentage of lesions as well as leakage per lesion. Increased intake of omega-3 long-chain polyunsaturated fatty acids (*ω*-3 LCPUFAs), endogenous agonists of PPARs [62], is associated with attenuation of pathologic retinal and choroidal angiogenesis [63]. More recently, SanGiovanni and his colleagues found that DNA sequence variation in PPAR *γ* coactivator 1 alpha, a gene encoding a coactivator of the *ω*-3 LCPUFAs-sensing PPAR-retinoid X receptor (RXR) transcription complex, may influence neovascularization in AMD [64]. The results suggest that multiple constituents (ligands and transcriptional coactivators) of the PPAR-RXR system may influence pathogenic processes in CNV. There is evidence that dysfunction of RPE around macula area may be responsible for the development of AMD [58, 65]. One of the most important functions of RPE is phagocytic uptake and degradation of photoreceptor outer segments [64]. A study showed that specific phagocytosis of photoreceptor outer segments by RPE cells selectively activates expression of PPAR *γ*, suggesting that PPAR *γ* may play an important role in the photoreceptor renewal process [66]. Oxidative stress is a major risk factor causing RPE cell degeneration. A number of studies have shown that RPE might be the prime target for oxidative stress and PPAR *γ* is implicated in the oxidative stress response [25]. In cultured human primary RPE cells and/or ARPE-19 cells, troglitazone and 15d-PGJ2 can protect cells from oxidative stress induced by t-butylhydroperoxide or H_2_O_2_ [67–69]. Other PPAR *γ* agonists, rosiglitazone [67, 69], pioglitazone [69], ciglitazone [68], AGN195037 [67], azelaoyl PAF [68], LY171883 [68], and WY14643 [68], however, are not effective. To determine whether the cytoprotective effects of troglitazone and 15d-PGJ2 were mediated by PPAR *γ*, PPAR *γ* expression was knocked down using RNA interference. In the cells lacking PPAR *γ* expression, troglitazone's protective effect was greatly blocked [69], while 15d-PGJ2's protective activity was not affected [67]. These results indicate that the cytoprotective effect of troglitazone is mediated by PPAR *γ* but the effect of 15d-PGJ2 is independent of PPAR *γ* activity, and PPAR *γ* agonists can have differential effects on RPE survival in response to oxidative stress.

#### 2.2.2. PPAR *γ* and DR

Diabetic retinopathy (DR) remains as the leading cause of blindness among working age individuals in developed countries, which is one of the most common microvascular complications of diabetes. TZDs, synthetic PPAR *γ* agonists, in addition to increasing insulin sensitivity and regulating lipid metabolism [70, 71], may also exert anti-inflammatory, antiatherogenic, neuroprotective, and antioxidative effects [72–75]. Because of these beneficial effects, they may have therapeutic potential in diabetic microvascular complications such as DR.

In vitro and in vivo experiments have demonstrated that TZDs may provide retinal microcirculatory stability [76–78], attenuate pathological retinal microvessel formation [79], inhibit the fibrotic change of RPE cells [80], and also prevent retinal neuronal damage [81] in diabetic and ischemic retinopathy. Recently, a study showed that pioglitazone might improve impaired insulin signaling in the diabetic rat retina [82]. Murata et al. [83] illustrated that TZDs may have the potential to inhibit the progression of DR. In vitro, they found that troglitazone and rosiglitazone could inhibit the proliferation of retinal endothelial cell and tube formation induced by VEGF. Meanwhile, using the oxygen-induced ischemia model of retinal neovascularization in neonatal mice they showed that intravitreous injection of troglitazone and rosiglitazone could inhibit development of retinal neovascularization. To support these experimental evidences, a clinical study showed that the progression from nonproliferative DR to proliferative DR over 3 years occurred in 19.2% in the rosiglitazone group and 47.4% in the control group, suggesting that rosiglitazone may delay the onset of proliferative DR [84]. However, there are some adverse effects of TZDs that have been reported. Several clinical studies showed that TZDs increased the risk of macular edema [32–35]. Other studies found that administration of pioglitazone [36] and troglitazone [37] significantly increased plasma VEGF expression in diabetic patients which increased risk of diabetic macular oedema (DME) and promoted the progression of DR. The relationship between TZDs and DME is still controversial. Some authors reported that they did not detect fluid retention in the macula or subclinical DME under TZDs treatment [85, 86]. Further clinical and experimental studies are urgently required.

Apart from these synthetic PPAR *γ* agonists, herbal and traditional natural medicines, such as* Astragalus membranaceus*,* Pueraria thomsonii* [87],* Swietenia mahagoni* [69], Korean red ginseng [59], Dan-shao-hua-xian formula [88–90], and Turmeric [91–94], have shown the potential effect in the modulation of DR through PPAR *γ* activation. Tom Huang's group summarized the current studies on herbal or traditional medicine associated with PPAR *γ* activation and the possible mechanisms relevant to the management of DR [8]. They confirmed that plant-derived PPAR *γ* activators could provide an alternative or combination therapy to prevent or delay the progression of DR.

#### 2.2.3. PPAR *γ* and Retinal Neuroprotection

It is well known that PPAR *γ* has neuroprotective effects in central nervous system (CNS) [95]. Several studies indicated that PPAR *γ* agonists could prevent or attenuate the process of neurodegenerative diseases in Parkinson's disease [96], Alzheimer's disease [97], and amyotrophic lateral sclerosis [98]. Various PPAR *γ* agonists (e.g., troglitazone, rosiglitazone, and pioglitazone) have shown beneficial effects in animal models of cerebral ischemia/reperfusion injury, ischemic stroke, intracerebral hemorrhage, traumatic brain injury, and spinal cord injury by attenuating neuronal cell death in the injured CNS [99–103]. Using a rat model of optic nerve crush (ONC), our research group demonstrated that PPAR *γ* activation is beneficial in retinal neuroprotection [104]. We found that PPAR *γ* was upregulated in rat retina after ONC and most of PPAR *γ* immunoreactive cells colocalized with Müller cells. Intraperitoneal injection of pioglitazone significantly enhanced the number of surviving retinal ganglion cells (RGCs) and inhibited RGCs apoptosis induced by ONC. But these neuroprotective effects were abrogated in the presence of PPAR *γ* antagonist GW9662. In addition, pioglitazone attenuated Müller cell activation after ONC. In coincidence with our results, Zhang et al. reported the protective effect of pioglitazone on the rat retina after ischemia/reperfusion injury [105]. They found that pioglitazone could inhibit activation of the glia cells, prevent cell apoptosis, and protect the retina from subsequent cellular damage caused by the retinal I/R. In vitro, other two PPAR *γ* ligands, 15d-PGJ2 and troglitazone, also appeared to protect RGC-5 cells against glutamate-induced cytotoxicity. To understand the more specific mechanisms of PPAR *γ*-based neuroprotection in retina, future studies would be needed.

### 2.3. PPAR *γ* and Other Ocular Diseases

PPAR *γ* has been found to be associated with thyroid eye disease (TED), Graves' ophthalmopathy (GO), or thyroid-associated orbitopathy (TAO), an autoimmune eye condition that is often seen with thyroid disease. The expression of PPAR *γ* was significantly increased in orbital tissue samples from patients with GO compared with normal orbital tissue [106, 107]. PPAR *γ* may play divergent roles in the process of the disease, both attenuating and promoting disease progression. PPAR *γ* activation is critical to adipogenesis, making it a potential culprit in the pathological fat accumulation associated with TED or GO [108]. Downregulation of PPAR *γ* could reduce adipogenesis [109]. Starkey reported that a male type 2 diabetic patient, treated with pioglitazone, experienced rapid exacerbation of his TED, which had been stable and inactive for more than 2 yr. In his in vitro experiments, by isolating and culturing preadipocytes from TED orbits, he demonstrated that the PPAR *γ* agonists resulted in a 2- to 13-fold increase, and a PPAR *γ* antagonist produced a 2- to 7-fold reduction in adipogenesis [110]. Consistent with this finding, sodium diclofenac, another antagonist of PPAR *γ*, also appeared to have efficacy in the treatment of GO [111]. However, PPAR *γ* also has anti-inflammatory activity. Pioglitazone and rosiglitazone have been found to inhibit TGF*β*-induced, hyaluronan-dependent, T cell adhesion to orbital fibroblasts, suggesting that they could inhibit intense inflammation and might be useful in treating TED [112]. Thus, if PPAR *γ* function is to be targeted as a TED therapeutic, PPAR *γ* modulators with selective activities would be required.

PPAR *γ* expression has been already studied in several tumors, and most studies implicate a protective effect of PPAR *γ* activation in tumors [113–116]. A recent study showed that PPAR *γ* was predominantly expressed in the cytoplasm of uveal melanoma tumor cells, suggesting that it might play an important role in the progression of uveal melanoma [117]. However, further studies are warranted to shed more light on a possible protective role of PPAR *γ* in this tumor.

Since PPAR *γ* has been shown to have the potential to treat autoimmune diseases [118–120], a murine model of experimental autoimmune uveoretinitis (EAU) was established to explore the efficacy of PPAR *γ* on endogenous uveitis. Intravenous injection of pioglitazone before and after the onset of EAU significantly reduced disease severity, suppressed intraocular concentrations of TNF-*α* and IL-6, and increased CD4(+)Foxp3(+) regulatory T cells and CD4(+)CD62L(high) naïve T cells in draining lymph nodes [121].

## 3. Significance and Future Prospects

TZDs (e.g., pioglitazone, troglitazone, and rosiglitazone) and 15d-PGJ2, as the existing therapeutic agents targeted to effect PPAR *γ*, may be the novel adjuvants in the treatment of ocular diseases. Pioglitazone and 15d-PGJ2 may inhibit corneal neovascularization and scar formation in cornea alkali burn injury model and VEGF-induced cornea angiogenesis and exert a therapeutic effect in dry eye mice. Rosiglitazone may represent a new strategy for inhibiting excessive bleb scarring in the cornel and conjunctiva after laser ablation and glaucoma filtering surgery. In retinal diseases troglitazone and rosiglitazone may attenuate the progression of AMD and DR in vivo. A clinical study suggested that rosiglitazone may delay the onset of proliferative DR. Pioglitazone and troglitazone also showed the neuroprotective effects in retina.

In summary, various experimental studies and several clinical studies have provided evidences that PPAR *γ* may emerge as a potential target for drugs that might be used in the treatment of ocular diseases in which PPAR *γ* activities play a key role in disease pathology. However, the complexity of PPAR *γ* activation not only provides beneficial effects but also introduces risks from undesirable side effects. Extensive preclinical and clinical trials are needed to establish the efficacy and to prove the safety of these drugs for the treatment of ocular diseases.

## Figures and Tables

**Table 1:** Ligands for PPAR *γ*.

Ligand class	Compounds	References
Endogenous agonists	15d-PGJ2,	[122–125]
	13-Hydroxyoctadecadienoic acid (13-HODE),	[122]
	9-hydroxyoctadecadienoic acid (9-HODE),	[122]
	15-hydroxyeicosatetraenoic acid (15-HETE),	[123]
	nitrolinoleic acid	[126]

Synthetic agonists	Pioglitazone, troglitazone, rosiglitazone, ciglitazone, TZD18,	[127–129]
	JTT-501, CDDO, SB-219994, SB-219993, GW2331, GW0072,	[130–133]
	5-ASA, PAT5A, TAK-559, GW7845, GW1929, LG10074,	[122, 134–137]
	indomethacin, ibuprofen, flufenamic acid, conjugated linoleic acid,	[123, 138–140]
	L-764406, L-796449, LY-510929, LY-465608, AD-5061, AD-5075,	[141–144]
	KRP-297/MK-0767, MCC555, ragaglitazar, farglitazar, diclofenac	[145–148]

Antagonists	GW9662, CDDO-Me, BADGE, PD068235, SR-202	[149, 150]
